# UPLC-Q–TOF–MS, network analysis, and molecular docking to investigate the effect and active ingredients of tea-seed oil against bacterial pathogens

**DOI:** 10.3389/fphar.2023.1225515

**Published:** 2023-09-07

**Authors:** Yan Duan, Li-Juan Zhao, Yan-Hui Zhou, Qi-Zhi Zhou, Ai-Qing Fang, Yu-Ting Huang, Yuan Ma, Zhi Wang, Yu-Ting Lu, Yu-Ping Dai, Shun-Xiang Li, Juan Li

**Affiliations:** ^1^ School of Pharmacy, Hunan University of Chinese Medicine, Changsha, China; ^2^ Hunan Amazing Grace Biotechnology Co, Ltd, Changsha, China; ^3^ Hunan Engineering Technology Research Center for Bioactive Substance Discovery of Chinese Medicine, Changsha, China; ^4^ Hunan Province Sino-US International Joint Research Center for Therapeutic Drugs of Senile Degenerative Diseases, Changsha, China

**Keywords:** tea-seed oil, antibacterial effect, methanol extraction, UPLC-Q-ToF-MS, network pharmacology, molecular docking

## Abstract

**Object:** This research intended to probe the antibacterial effect and pharmacodynamic substances of Tea-Seed Oil (TSO) through the use of ultra-performance liquid chromatography-quadrupole time-of-flight mass spectrometry (UPLC-Q-TOF/MS) analysis, network analysis, and molecular docking.

**Methods:** The major chemical components in the methanol-extracted fractions of TSO were subjected to UPLC-Q-TOF-MS. Network pharmacology and molecular docking techniques were integrated to investigate the core components, targets, and potential mechanisms of action through which the TSO exert their antibacterial properties. To evaluate the inhibitory effects, the minimum inhibitory concentration and diameter of the bacteriostatic circle were calculated for the potential active ingredients and their equal ratios of combinatorial components (ERCC) against *Escherichia coli*, *Staphylococcus aureus*, *Pseudomonas aeruginosa*, and *Candida albicans*. Moreover, the quantification of the active constituents within TSO was achieved through the utilization of high-performance liquid chromatography (HPLC).

**Results:** The methanol-extracted fractions contained a total of 47 chemical components, predominantly consisting of unsaturated fatty acids and phenolic compounds. The network pharmacology analysis and molecular docking analysis revealed that various components, including gallocatechin, gallic acid, epigallocatechin, theophylline, chlorogenic acid, puerarin, and phlorizin, have the ability to interact with critical core targets such as serine/threonine protein kinase 1 (AKT1), epidermal growth factor receptor (EGFR), a monoclonal antibody to mitogen-activated protein kinase 14 (MAPK14), HSP90AA1, and estrogen receptor 1 (ESR1). Furthermore, these components can modulate the phosphatidylinositol-3-kinase protein kinase B (PI3K-AKT), estrogen, MAPK and interleukin 17 (IL-17) signaling pathways, hereby exerting antibacterial effects. *In vitro* validation trials have found that seven components, namely gallocatechin, gallic acid, epigallocatechin, theophylline, chlorogenic acid, puerarin, and phloretin, displayed substantial inhibitory effects on *E. coli, S. aureus*, *P. aeruginosa*, and *C. albicans*, and are typically present in tea oil, with a total content ranging from 15.87∼24.91 μg·g^−1^.

**Conclusion:** The outcomes of this investigation possess the possibility to expand our knowledge base concerning the utilization of TSO, furnish a theoretical framework for the exploration of antibacterial drugs and cosmetics derived from inherently occurring TSO, and establish a robust groundwork for the advancement and implementations of TOS products within clinical settings.

## 1 Introduction

Tea seed Oil (TSO), is extracted from the seeds of *Camellia oleifera* Abel. It is rich in unsaturated fatty acids, theasaponins, tea polyphenols, vitamins, squalene, and carotenoids, and is typically used as food (Zhang et al., 2022; Hu et al., 2022). In addition, TSO possesses medicinal value, is widely used as an injection material and ointment base, and is currently indexed in the Chinese Pharmacopoeia (2020 version) (Xu et al., 2021). Modern pharmacological studies have revealed that TSO possesses antibacterial (Duan et al., 2021; Zhang et al., 2021), anti-inflammatory (Duan et al., 2021), and antioxidant effects (Wang et al., 2022a) and is clinically used to treat diseases such as dermatitis, pressure ulcers, burns, neonatal red glut, diaper dermatitis, and pediatric diaper rash induced by bacterial fungal infections with excellent efficacy (Zou and Yi, 2013; Cheng et al., 2014). TSO has been used as a matrix excipient in the formulation of cosmetic and medicinal products due to its favorable attributes, including antimicrobial properties and compatibility with the skin (Wang and Wu, 2018), as well as its ability to enhance the penetration of fat-soluble substances (Wang et al., 2022b; Duan et al., 2022). Nevertheless, the specific antibacterial components of TSO have yet to be identified, impeding its widespread application in the fields of medicine and cosmetics (Shi et al., 2020). Consequently, a comprehensive and rigorous investigation into the chemical composition and pharmacological effects of TSO is imperative to furnish substantial scientific evidence for its effective utilization and further development.

Chemical composition and pharmacological activity of TSO vary depending on the method used for extraction, including mechanical pressing and solvent extraction. A prior study found that solvent extraction of TSO produced a superior antibacterial effect than pressing. Interestingly, we also found that the pre-treatment of medicinal TSO, including deacidification, decolorization, and deodorization processes, considerably influences its antibacterial activity, especially the deacidification (Duan et al., 2022). This indicates that the composition of tea oil components is closely associated to the extraction methodology, and also affects its pharmacological properties. In the decidification procedure, the primary purpose is to remove free fatty acids and enhance the stability of TSO. However, many acidic biological small molecules such as phenolic acids and organic acids are also removed, which may be the main reason for their diminished antibacterial activity. Therefore, the antibacterial effect of TSO can be researched by analyzing the changes in its composition during deacidification. In this study, employed ultra-performance liquid chromatography-quadrupole time-of-flight mass spectrometry (UPLC-Q-TOF-MS) to qualitatively ascertain the primary chemical constituents in the methanol-extracted fractions of TSO. Additionally, network pharmacology and molecular docking techniques were integrated to investigate the fundamental components, targets, and potential mechanisms of action through which the functional constituents exert their antibacterial properties. Drawing from these preliminary findings, the antibacterial effects of potential active ingredients and their equal ratios of combinatorial components (ERCC) and the contents of active ingredients from TSO have been researched and determined. We anticipate that this study will provide guidelines for the development, utilization, and clinical application of TSO.

## 2 Materials and methods

### 2.1 Materials and reagents

In all cases, the purity of the standards exceeded 98%, and Epigallocatechin (Cat. K22N9R75650), gallocatechin (cat. A25GB156582), gallic acid (purity 98.0%, lot Y20J12Q138091), phloretin (Cat. Y05M11Y17319), and puerarin (cat. S02M9B45875) were purchased from Shanghai Yuanye Biotechnology Co., Ltd. (Shanghai, China). Chlorogenic acid (Cat. CHB170713) was purchased from Chengdu Kloma Biotechnology Co., Ltd. (Chengdu, China). Theophylline (Cat. DB19-SP4V) was purchased from the National Institute for Food and Drug Control. The remaining reagents were of analytical grade and supplied by Sinopharm Chemical Reagent Co., Ltd. (Shanghai, China).

Amoxicillin Capsules were purchased from Zhuhai Lianbang Pharmaceutical Co. Ltd. (Cat. 90305002), and flupionate capsules were purchased from Henan Tianfang Pharmaceutical Co., Ltd. (Cat. 190410132). Fluconazole capsules were purchased from Guangdong Hushu Pharmaceutical Co., Ltd. (cat. 202000204), and nutrient broth medium agar was purchased from Qingdao High Science Park Hybo Biotechnology Co., Ltd. (Cat. 20191126). The martin-modified medium agar was purchased from Guangdong Huankai Microbial Technology Co., Ltd. (Cat. 1073301).

### 2.2 Sample preparation and standard solution

**FIGURE 1:**
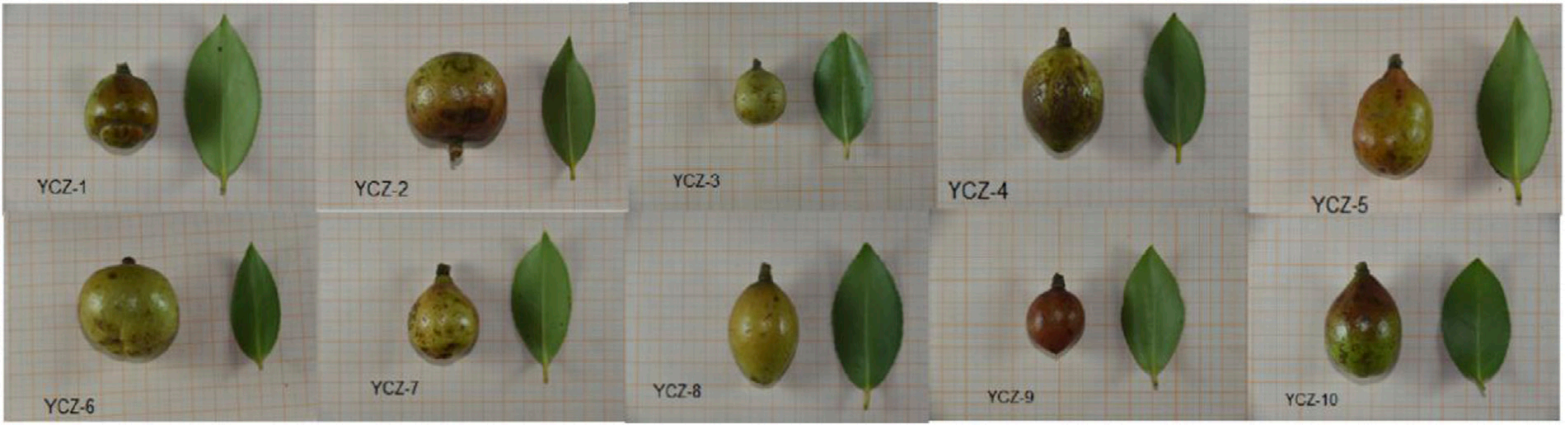
The fruits and leaves of *Camellia oleifera* Abel. Each of the YCZ-1—YCZ-10 represents a sample of *Camellia oleifera* seeds from a different species.

The *Camellia oleifera* seeds were collected from Xiangchun Planting Base (113.5°E, 28.3°N) in Liuyang, Hunan Province in October 2019. The samples were deposited at Herbarium, College of Pharmacy, Hunan University of Chinese Medicine (Changsha, China), voucher number is YCZ-1, YCZ-2, YCZ-3, YCZ-4, YCZ-5, YCZ-6, YCZ-7, YCZ-8, YCZ-9 and YCZ-10, respectively. (Figure 1). *Camellia oleifera* seeds (10 g) were ground into powder and sieved through a 60-mesh sieve, then added with 50 mL of n-hexane for reflux extraction at 90°C for 1 h. The clear solution is filtered and collected, and the residue is then added with 50 mL of n-hexane for repeat the previous steps, merge the clear solution, and steam it under a temperature of 50°C and a vacuum of 0.1 MPa to obtain TSO.

Prior to the experiment, the preparation method was optimized to determine the optimal conditions. TSO samples (10.0 g) were weighed and ultrasonicated for 10 min in 30 mL methanol. The supernatant was concentrated under reduced pressure at 50 C to a solvent-free drop, weigh and calculate the yield of methanol-extracted TSO (3.69%), then a suitable amount of methanol was added to dissolve it to 10 mL. For each standard component, methanol was used to dissolve the stocks, which were further diluted with methanol to reach a suitable concentration. An appropriate amount of single standard substance configuration equal-ratio combinatorial components (ERCC), in which the ratios of gallocatechin, gallic acid, epigallocatechin, theophylline, chlorogenic acid, puerarin, and phlorizin were 14:6:13:4:1:1:1. Before use, all solutions were stored at −4°C in the dark.

### 2.3 UPLC-Q-TOF-MS analysis of the chemical ingredients of tea-seed oil

#### 2.3.1 UPLC-Q-TOF-MS qualitative analysis

A Waters AcquityTM Ultra Performance LC system coupled with a Xevo™ QTof mass spectrometer (Waters, United States) was used for UPLC-Q-TOF-MS analysis, and Masslynx 4.1 was used to process the data.

Based on the reference (Liu et al., 2021), the chromatographic detection reference method was modified, acetonitrile (A) and water (B) (both including 0.1% formic acid, v/v) were used as the mobile phase in an ACQUITY UPLC BEH C_18_ column (2.1 mm × 100 mm, 1.8 μm) under 30 C chromatographic conditions. The elution procedure was performed as follows: 1) 0–2 min,30%–50% A; 2) 2–8 min,50%–70% A; 3) 8–16 min,70%–80% A; 4) 16–28 min,80%–90% A; 5) 28–30 min, 90% A. Flow rate was maintained at 0.3 mL·min^-1^, and volume of injected sample was 2 μL.

Detection was performed in both electrospray positive and negative ion modes using magnetic resonance elastography (MRE) scanning and corrected for accurate mass using the ESI-L Low Concentration Tuning Mix (G1969-8500). The primary full scan mass range was from m/z 100–1200 with a resolution of 30,000, except the solvent gas, which was N_2_; the drying gas flow rate was 6.8 L·min^−1^; capillary voltage was 4.0 kV; fragment voltage was 110 V; sheath gas temperature was 350 C. Secondary mass spectral data were acquired using dependent scanning, with the top three strengths selected for induced collisional dissociation based on the primary scan.

#### 2.3.2 Ingredient identification and analysis

A sample solution of TSO was analyzed using the UPLC-Q-TOF-MS detection system. Based on these data, the elements and molecular formulas of the ingredients in TSO were deduced. Please refer to websites, such as ChemSpider, ChemicalBook, and TCMSP, to clarify the structural formula of the compound.

### 2.4 Exploration of the antibacterial effect of tea-seed oil in the treatment by network pharmacology

The action targets of the primary components of TSO were retrieved and predicted using the TCMSP (https://old.tcmsp-e.com/tcmsp.php), PubChem, and Swiss Target Predict websites and imported into the Uniport online website to convert the targets into corresponding gene ID. The Genecards, DisGeNET website was used to locate disease gene targets using the keywords “bacteriostat” and “bacterial infection,” which were filtered and identified. Through Venny2.1.0 intersection targets, the common targets after intersection were imported into the STRING database to construct a protein interaction network diagram, and the common targets of R software were used for Gene Ontology (GO) enrichment analysis and Kyoto Encyclopedia of Genes and Genomes (KEGG) enrichment analysis to draw GO and KEGG plots.

### 2.5 Molecular docking between the potential compounds and the core predicted targets

Genes with Betweenness greater than 20 and degree greater than 10 were selected as the core target genes. Docking between the core target protein and small molecules, the main active ingredients of TSO, was performed using LeDock software, and the docking results were visualized and modified using PyMOL software.

### 2.6 Antibacterial experiment

#### 2.6.1 Determination of minimal inhibitory concentration (MIC)

All model bacterial strains, *Escherichia coli* (*E*. *coli*, ATCC NO. 44102), *Staphylococcus aureus* (*S*. *aureus*, ATCC NO. 26003), *Pseudomonas aeruginosa* (*P*. *aeruginosa*, ATCC NO. 90271), and *Candida albicans* (*C*. *albicans*, ATCC NO. 10231), were purchased from Nanjing Bianzhen Biotechnology Co., Ltd. (Nanjing, China). According to (Li et al., 2022), MIC testing was performed using the broth microdilution method. *E*. *coli, S*. *aureus,* and *P*. *aeruginosa* (1 × 10^5^ CFU·mL^−1^∼5 × 10^5^ CFU·mL^−1^) were inoculated in a nutrient broth medium, and *C*. *albicans* was inoculated in a martin-modified medium; the samples were also diluted in a twofold gradient from 500 μg·mL^−1^–3.9 μg·mL^−1^, bacteria solutions and the diluted samples were added in a ratio of 1:10. 96-well plates were incubated at 37 C for 24 and 48 h, and absorbance at 600 nm was measured using a microplate reader. In a triplicate experiment, the lowest concentration that could inhibit bacterial growth by 100% was defined as the minimum inhibitory concentration.

#### 2.6.2 Determination of the diameter of the bacteriostasis circle

The diameters of the bacteriostatic circles of the samples were determined using the hole-punch method referred to and modified by Mohamed (Mohamed et al., 2022). Briefly, the bacteria were evenly distributed on the surface of the agar plate, and a well with an inner diameter of 6 mm was punched into the middle of the agar plate. After half an hour of incubation at 37°C, 0.1 mL of the sample solution dissolved in ethanol was added to each well. Ethanol was used as a negative control, amoxicillin as a positive control for *S. aureus*, flupironic as a positive control drug in *E. coil* and *P. aeruginosa*, and fluconazole as a positive control for *C. albicans*. After 24–48 h of incubation, the inhibition zone formed around the cylinder was measured, and the test was repeated three times.

### 2.7 Determination of the contents of active ingredients from tea-seed oil

A slight modification was made to the chromatographic detection method using the reference (Chen, 2018), methanol (A) and water (B) (both including 0.2% formic acid, v/v) were used as the mobile phase in a Venusil XBP C_18_ column (4.6 mm × 250 mm, 5 μm) under 30 C chromatographic conditions. The elution procedure was performed as follows: 1) 0–4 min, 32%–30% A; 2) 4–9 min, 30% A; 3) 9–12 min, 30%–70% A; 4) 13–20 min, 70% A; 5) 20–23 min, 70%–32% A. Flow rate was maintained at 1.0 mL·min^−1^, detection wavelength was set at 280 nm, and volume of injected sample was 5 μL.

### 2.8 Statistical analyses

Statistics were performed using SPSS 25.0 software and data are presented as mean ± SD based on three independent experiments. *p* < 0.05 indicates a significant difference.

## 3 Result

### 3.1 UPLC-Q-TOF-MS analysis of the chemical ingredients of tea-seed oil

**FIGURE 2:**
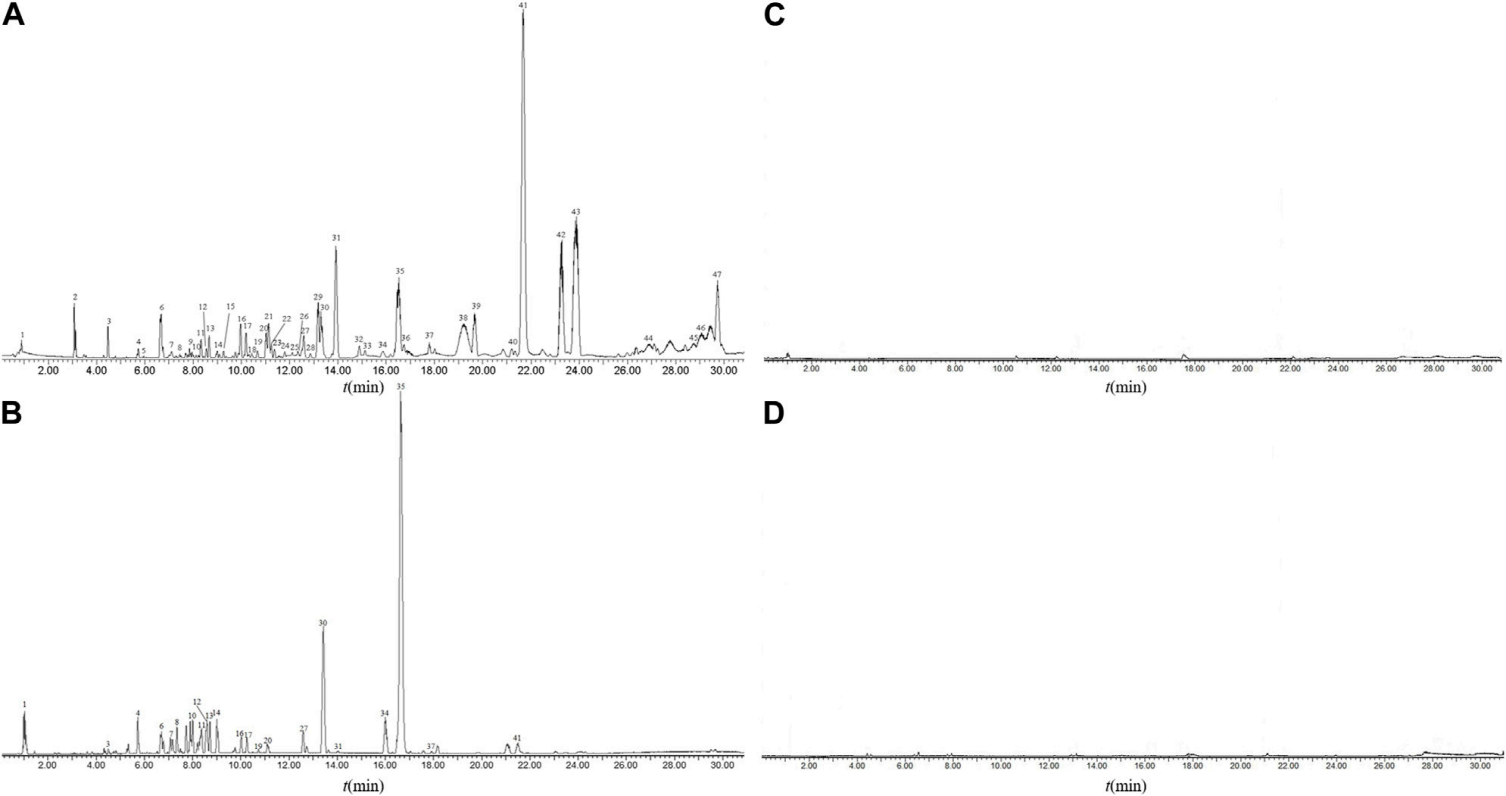
Base peak chromatogram (BPC) of sample solution. BPC of TSO-M sample solution in the positive ion mode **(A)** and the negative ion mode **(B)**, BPC of blank sample solution in the positive ion mode **(C)** and the negative ion mode **(D)**.

UPLC-Q-TOF-MS analysis of the methanol extraction site of tea seed oil (TSO-M) was performed, and the BPC of sample under positive and negative ion detection modes were superimposed and compared, as displayed in Figure 2. The number of compounds detected in the positive ion mode was greater than that of all compounds detected in the negative ion mode, and 47 compounds were identified in TSO-M, as shown in Table 1, which primarily contained unsaturated fatty acids such as oleic, linoleic, palmitic, stearic, behenic, and arachidic acids, as well as phenolic compounds such as phloretin, puerarin, chlorogenic acid, catechin derivatives, tocopherols, and quinic acid. It also contains components such as theophylline, lutein, and carotene.

**TABLE 1:** Ingredients identified in TSO based on UPLC-Q-TOF-MS (positive ion mode).

No.	Peak appearance	Molecular formula	Theoretical value(m/z)	Value(m/z)	Deviation	Ion species<	Ion fragment (m/z)	Ingredient name	Ref
	Time (min)				ΔPPM				
1	0.89	C_20_H_20_O_5_	341.3706	341.3702	−1.2	[M + H]^+^	323.2467, 267.1429	(S)-4′,5,7-Trihydroxy-3′-prenylflavanone	Adib et al. (2008)
2	3.07	C_14_H_23_NO_2_S	270.5143	270.5148	1.8	[M + H]^+^	256.2643, 158.9611	Octadecylamine	Varshosaz et al. (2012)
3	4.46	C_17_H_34_O_2_	271.4506	271.4501	−1.8	[M + H]^+^	381.2689, 351.2423	Ethyl pentadecanoate	-
4	5.76	C_15_H_14_O_7_	307.2707	307.2701	−2.0	[M + H]^+^	289.0931, 169.9784	(+)-Gallocatechin	Huang et al. (2019)
5	5.97	C_7_H_6_O_5_	171.1251	171.1257	3.5	[M + H]^+^	163.0752, 139.9847	Gallate	Huang et al. (2019)
6	6.69	C_18_H_33_NO	280.2563	280.2562	−0.4	[M + H]^+^	263.2389, 262.2281	Linoleamide	-
7	7.10	C_15_H_14_O_7_	307.2671	307.2678	2.3	[M + H]^+^	273.1668, 153.9638	(-)-Epigallocatechin	Huang et al. (2019)
8	7.68	C_17_H_33_NO_3_	300.2416	300.2416	0.0	[M + H]^+^	282.2429, 256.2562	Pentadecanoylglycine	-
9	7.78	C_22_H_38_O_10_	463.2427	463.2423	−0.9	[M + H]^+^	445.3412, 317.2141	xi-Linalool-3-rhamnopyranosyl-(1→6)-glucopyranoside	-
10	7.90	C_18_H_35_NO_3_	314.2632	314.2637	1.6	[M + H]^+^	296.2219, 268.0670	Palmitoylglycine	-
11	8.33	C_15_H_22_NO_4_	281.4462	281.4468	2.1	[M + H]^+^	263.2278, 171.1478	Feruloylcholine	Hu et al. (2022)
12	8.55	C_16_H_27_NO_2_S	298.3418	298.3416	−0.7	[M + H]^+^	279.2321, 165.1591	2-(4-Methyl-5-thiazolyl)ethyl decanoate	-
13	8.66	C_18_H_35_NO_2_	298.2674	298.2676	0.7	[M + H]^+^	279.2321, 237.1824	Palmitoleoylethanolamde	Hu et al. (2022)
14	8.94	C_18_H_37_NO	284.2828	284.2825	−1.1	[M + H]^+^	267.2694, 249.1845	Octadecanamide	Hu et al. (2022)
15	9.26	C_40_H_56_O	569.8710	569.8714	0.7	[M + H]^+^	527.3804, 415.2878	Flavochrome	Zhang et al. (2019)
16	9.97	C_22_H_44_O_2_	341.3384	341.3387	0.9	[M + H]^+^	323.2401, 267.1639	Butyl octadecanoate	Hu et al. (2022)
17	10.19	C_22_H_44_O_2_	341.5834	341.5839	1.5	[M + H]^+^	323.2392, 305.2653	Behenic acid	Hu et al. (2022)
18	10.43	C_20_H_42_NO_7_P	440.2170	440.2174	0.9	[M + H]^+^	422.3501, 299.2554	Lysophosphatidyl ethanolamine	-
19	10.66	C_15_H_15_NO_8_	338.2782	338.2785	0.9	[M + H]^+^	320.0872, 302.1842	2,8-Dihydroxyquinoline-beta-D-glucuronide	Zhang et al. (2021)
20	11.13	C_16_H_25_NO_3_	280.3275	280.3271	−1.4	[M + H]^+^	262.2243, 149.9331	N-[(4-Hydroxy-3-methoxyphenyl)methyl] octanamide	-
21	11.24	C_21_H_42_O_2_	327.5639	327.5633	−1.8	[M + H]^+^	309.2763, 291.2479	Heneicosanoic acid	Jia et al. (2021)
22	11.37	C_20_H_38_O_2_	311.2287	311.2282	−1.6	[M + H]^+^	270.2504, 186.9813	9Z-Eicosenoic acid	Jia et al. (2021)
23	11.54	C_16_H_32_O_2_	256.4240	256.4247	2.7	[M + H]^+^	239.1781, 221.6234	Palmitic acid	Hu et al. (2022)
24	11.93	C_18_H_34_O_2_	283.4656	283.4657	0.4	[M + H]^+^	253.2483, 242.2837	Oleic acid	Hu et al. (2022)
25	12.10	C_13_H_21_N_5_O_2_	181.1670	181.1675	2.8	[M + H]^+^	163.1000, 124.2000	Theophylline	Jwc et al. (2012)
26	12.23	C_17_H_34_O_2_	271.3256	271.3259	1.1	[M + H]^+^	255.2309, 237.2221	Methyl hexadecanoic acid	-
27	12.59	C_22_H_42_O_2_	339.3218	339.3213	−1.5	[M + H]^+^	283.2836, 265.2532	N-butyl Oleate	-
28	12.86	C_22_H_39_NO	334.3403	334.3401	−0.6	[M + H]^+^	316.2642, 293.2422	2,4,12-Octadecatrienoic acid isobutylamide	-
29	13.18	C_18_H_19_NO_4_	314.2625	314.2623	−0.6	[M + H]^+^	279.0953, 221.2274	Moupinamide	-
30	13.29	C_15_H_19_NO_9_	358.2130	358.2134	1.1	[M + H]^+^	340.2913, 298.2826	2-Methoxyacetaminophen glucuronide	-
31	13.91	C_20_H_37_NO_3_	340.2718	340.2712	−1.8	[M + H]^+^	322.2713, 294.0892	Oleoyl glycine	-
32	14.89	C_16_H_18_O_9_	355.3151	355.3154	0.8	[M + H]^+^	337.2721, 309.2791	Chlorogenic acid	Wang et al. (2015)
33	15.15	C_21_H_20_O_9_	417.3780	417.3785	1.2	[M + H]^+^	321.3133, 265.2515	Puerarin	Xu et al. (2021)
34	18.85	C_18_H_36_O_2_	285.4821	285.4825	1.4	[M + H]^+^	267.2713, 249.1825	Stearic acid	Hu et al. (2022)
35	16.51	C_18_H_34_O	267.2161	267.2165	1.5	[M + H]^+^	247.2422, 237.1845	9-Octadecenal	Jia et al. (2021)
36	16.74	C_15_H_14_O_5_	275.2769	275.2763	−2.2	[M + H]^+^	293.2845, 275.2733	Phloretin	Xu et al. (2014)
37	17.78	C_20_H_39_NO_3_	342.5229	342.5224	−1.5	[M + H]^+^	324.2989, 267.2684	Stearoyl glycine	-
38	19.22	C_29_H_50_O_2_	430.7161	430.7165	0.9	[M + H]^+^	413.3302, 387.3455	*α*-Tocopherol	Hu et al. (2022)
39	19.66	C_18_H_32_O_3_	297.2235	297.2233	−0.7	[M + H]^+^	279.0921, 263.2373	*α*-Dimorphecolic acid	Kuang et al. (2015)
40	21.33	C_20_H_40_O_2_	313.5300	313.5305	1.6	[M + H]^+^	280.0961, 251.2415	Arachidic acid	Hu et al. (2022)
41	21.69	C_16_H_18_O_8_	339.3109	339.3102	−2.1	[M + H]^+^	339.3402, 284.2978	3-*O*-*p*-Coumaroylquinic acid	Kuang et al. (2015)
42	23.28	C_17_H_34_O_2_	272.2156	272.2159	1.1	[M + H]^+^	253.2499, 242.2868	Heptadecanoic acid	Jia et al. (2021)
43	23.88	C_18_H_35_NO_2_	298.3451	298.3459	2.7	[M + H]^+^	280.0867, 266.2575	Palmitoyl ethanolamine	-
44	27.76	C_37_H_74_NO_7_P	676.5240	676.5247	1.0	[M + H]^+^	659.5351, 641.5497	PE(P-18:0/14:0)	-
45	28.74	C_40_H_58_O_5_	619.8185	619.8188	0.5	[M + H]^+^	603.5056, 601.5118	3,6-Epoxy-5,5′,6,6′-tetrahydro-b,b-carotene-3′,5,5′,6′- tetrol	-
46	29.07	C_37_H_71_O_8_P	675.1941	675.1953	1.8	[M + H]^+^	657.5038, 575.5024	PA(18:1(9Z)/16:0)	-
47	29.74	C_27_H_43_NO_3_	430.3224	430.3232	1.9	[M + H]^+^	409.3833, 395.3687	N-Oleoyl phenylalanine	-

### 3.2 Exploration of the antibacterial effect of tea-seed oil in the treatment by network pharmacology

#### 3.2.1 Target screening of active ingredients of tea-seed oil

The active ingredients in TSO, including gallocatechin, gallic acid, epigallocatechin, theophylline, chlorogenic acid, puerarin, and phlorizin, were screened for prediction targets using the TCMSP, PubChem, and Swiss target prediction websites, which were imported into the UniProt database for correction and then dereplicated, resulting in a total of 164 potential targets.

#### 3.2.2 Common targets and protein interaction network of antibacterial activity of tea-seed oil ingredients

**FIGURE 3:**
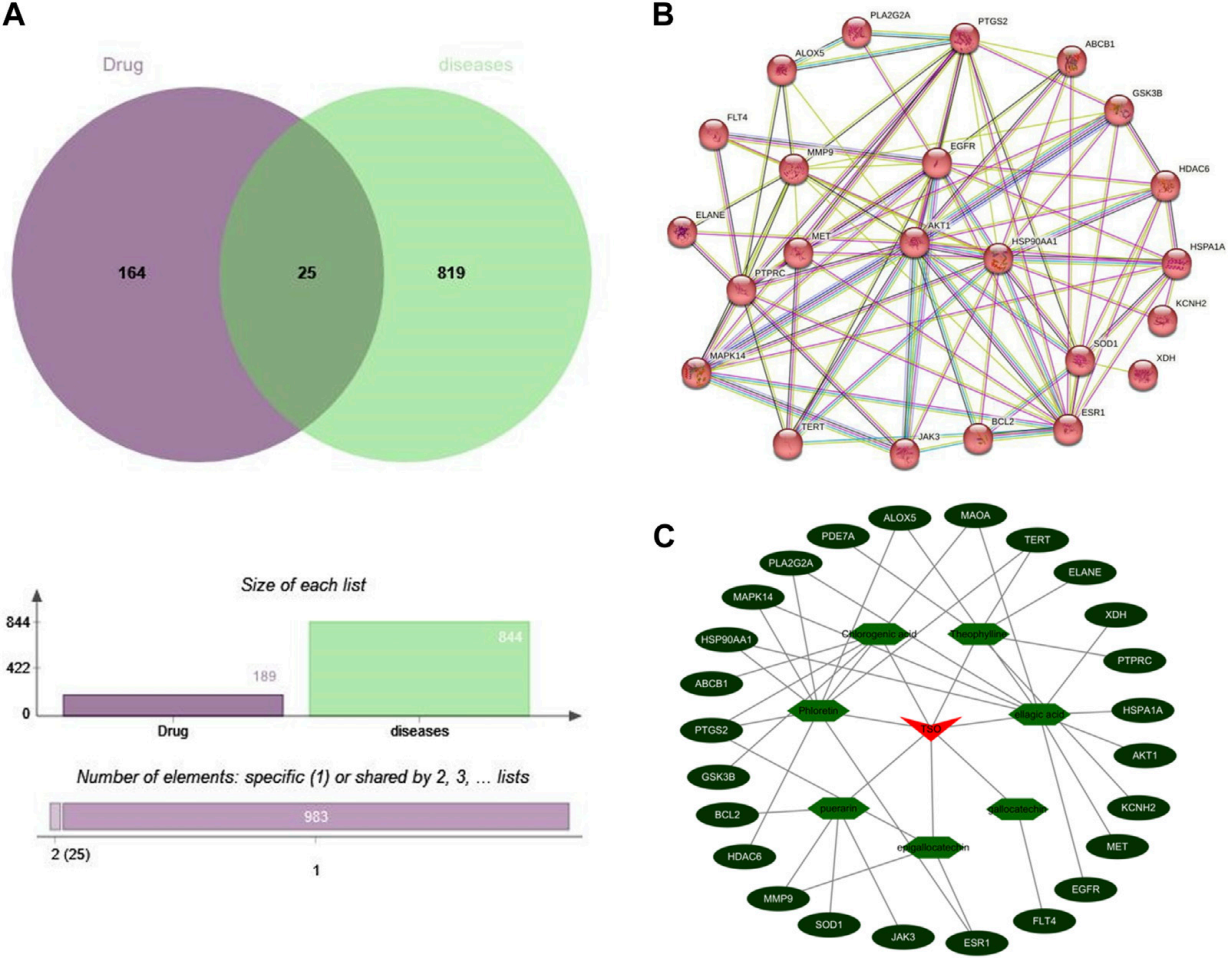
The prediction results of network pharmacology **(A)** Venn diagram of the active component targets of TSO and the targets related to antibacterial effect. **(B)** PPI network of the antibacterial effects of TSO. **(C)** The“drug-component-target”network of the antibacterial effects of TSO.

A total of 25 common targets were identified from the intersection of 164 potential principal component targets and 819 antibacterial targets (Figure 3A). These 25 common targets were imported into the STRING database to construct a PPI network, see Figure 3B. A “TSO-active ingredient-core target” network diagram was constructed using Cytoscape software by importing the active ingredients and their targets, see Figure 3C. The results revealed that there were several active ingredients corresponding to one target, and there was also one active ingredient acting on multiple targets, which embodied the effect characteristics between the active ingredients in TSO and the potential targets of the antibacterial effects.

#### 3.2.3 GO and KEGG pathway enrichment analysis

**FIGURE 4:**
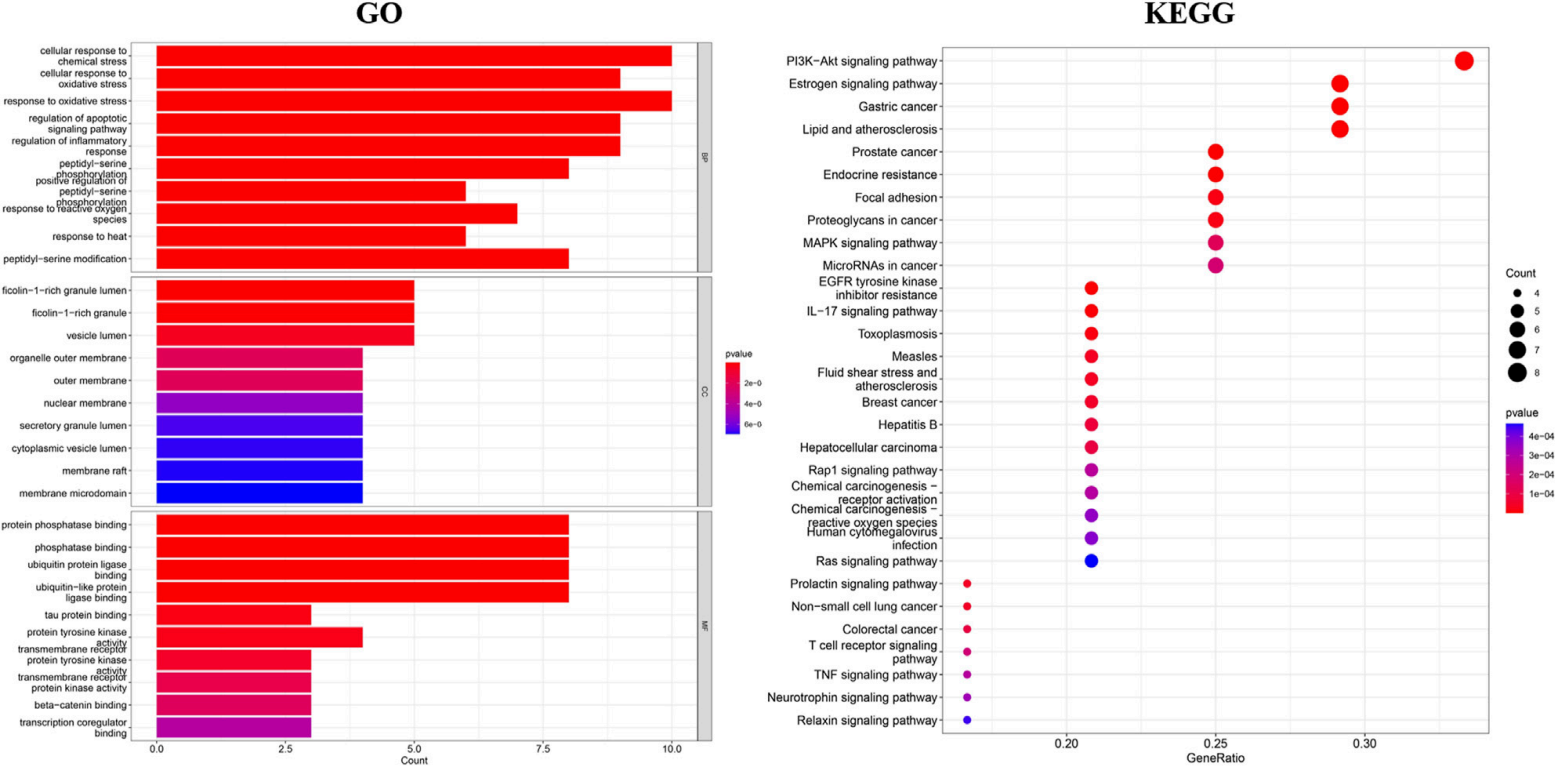
GO and KEGG enrichment analysis of the potential antibacterial targets of TSO.

**FIGURE 5:**
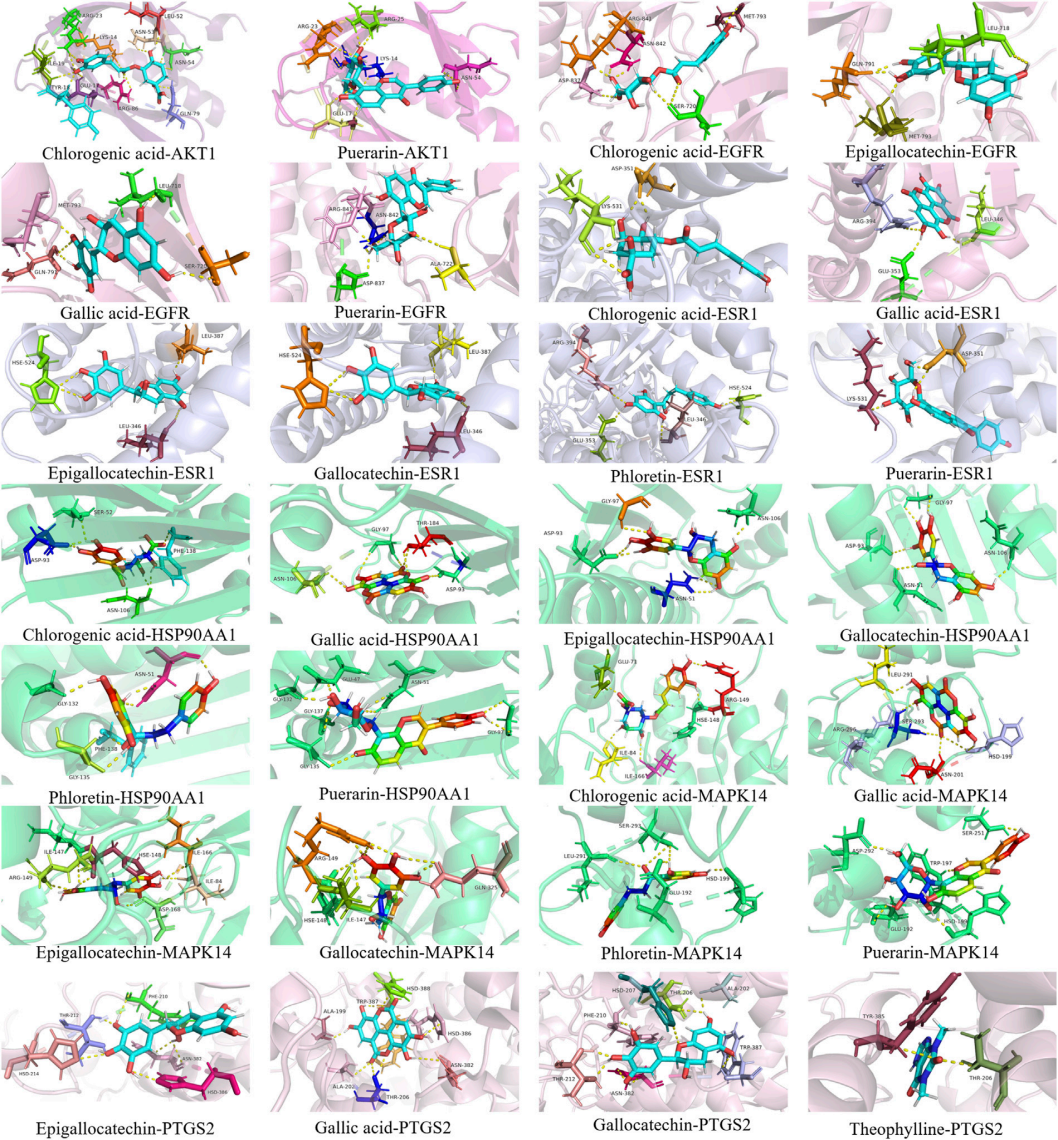
Molecular docking of the antibacterial core components and key targets of TSO.

GO enrichment analysis of common targets was performed using R software, in which biological pathway analysis resulted in a total of 1368 entries, principally related to the cellular stress response, regulation of apoptotic signaling pathways, and inflammatory signaling pathways. A comprehensive analysis was conducted on a total of 58 entries peretaining to the cellular composition, with a predominant focus on cellular vesicles, organelles, outer membranes, extracellular membranes, and nuclear envelopes. Additionally, a total of 111 entries were obtained for the analysis of molecular function, primarily centered around protein phosphatase binding, ubiquitin protein ligase binding, tyrosine kinase activity, and transcriptional regulation. The findings from this analysis suggest a potential correlation between the antibacterial effects of the major components of TSO and their ability to modulate signaling pathways, altering cellular reactions to external stress, and affecting protein modifications. The top 10 entries with the smallest *p*-values were selected as bar charts according to *p* < 0.05, as shown in Figure 4. KEGG pathway enrichment was screened with *p* < 0.05, and the top 30 ranked pathways were selected to create a bubble plot, as displayed in Figure 4. The results demonstrated that the enriched pathways included the phosphatidylinositol-3-kinase protein kinase B (PI3K-AKT), estrogen, MAPK, and interleukin 17 (IL-17) signaling pathways, implying that TSO components may exert antibacterial effects through multiple pathways and targets. Figure 5.

### 3.3 Results of molecular docking between the potential compounds and the core predicted targets

**FIGURE 6:**
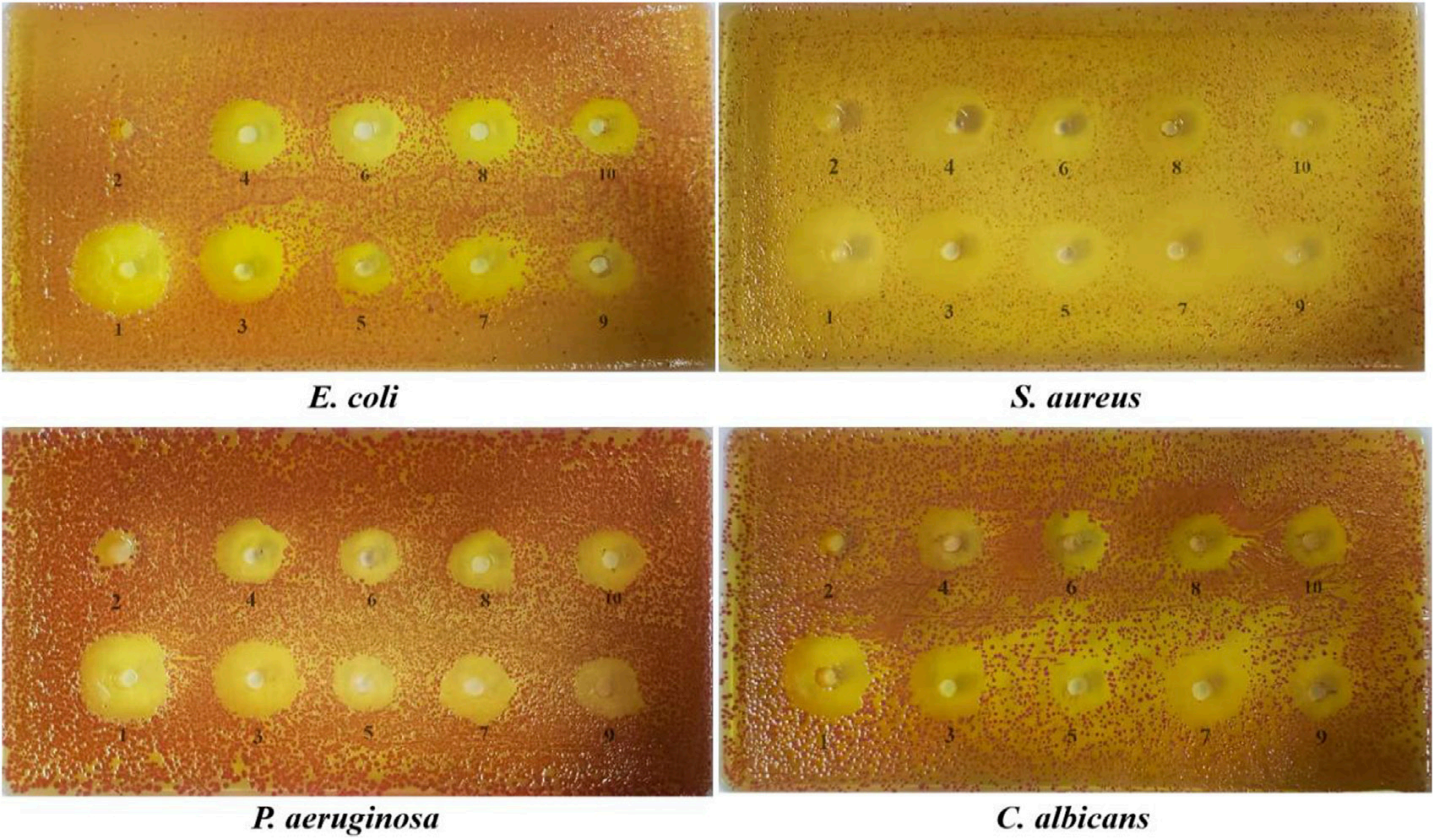
The diameter of the bacteriostasis circle of potential compounds.1: positive drug, 2: ethanol, 3: equal ratios combinatorial components, 4: chlorogenic acid, 5: theophylline, 6: gallic acid, 7: phloretin, 8: gallocatechin, 9: puerarin, 10: epigallocatechin.

Seven potential bacteriostatic components of camellia oil were selected for molecular docking with six core protein targets, and the strength of small-molecule protein binding was evaluated based on the magnitude of the absolute value of the binding energy. In overall, when the binding energy is lower than −4.25 kcal·mol^-1^ indicates some interaction between ligand small molecules and receptor proteins, −5.0 kcal·mol^-1^ or greater is reached between the two molecules, the binding activity is favorable. If −7.0 kcal·mol^-1^ or greater was achieved between the receptor and ligand, the conjugation activity was potent. The docking data were analyzed with a heat map, see Table 2, and the shades of color represent the strength of the correlation; the darker the color, the smaller the binding energy, and the stronger the binding effect, illustrating that some small molecules, such as gallocatechin, chlorogenic acid, epigallocatechin, ellaic acid, and puerarin, have better affinity with the core targets. Using the PyMOL software, we visualized the results obtained when small molecules were blended with proteins, as shown in Figure 6. Gallocatechin forms hydrogen bonds with amino acid residues ALA-202, THR-206, HSD-207, PHE-210, THR-212, ASN-382, and TRP-387 in PTGS2.

**TABLE 2:** Binding energy of core components and key targets of TSO.

Components	Binding energy/kcal·mol^-1^					
	AKT1	EGFR	ESR1	HSP90AA1	PTSG2	MAPK14
Chlorogenic acid	−5.94	−6.08	−7.08	−6.98	−8.04	−6.31
Ellgaic acid	−5.11	−5.72	−5.79	−5.9	−6.61	−5.71
Epigallocatechin	−5.08	−5.61	−7.18	−6.04	−7.18	−6.03
Gallocatechin	−5.22	−5.47	−6.94	−5.76	−7.00	−5.86
Phloretin	−5.10	−5.09	−6.5	−5.88	−6.75	−6.04
Puerarin	−5.62	−5.81	−5.77	−7.14	−7.49	−5.94
Theophylline	−4.47	−4.71	−4.68	−4.64	−5.23	−4.78

### 3.4 Examination of the antibacterial effect of potential compounds from tea-seed oil

#### 3.4.1 The diameter of the bacteriostasis circle of potential compounds

The diameters of the bacteriostatic circles for the potential compounds are shown in Figure 6 and Table 3. The antibacterial activity of each sample was measured by the size of the bacteriostatic circle. The results showed that the blank control (ethanol) had no antibacterial effect, indicating that the solvent had no interference, and the seven components and equal component groups had some inhibitory effect on the four bacteria. The inhibition zones of ERCC and phlorizin were relatively larger than those of the other components, implying that ERCC and phlorizin have better antibacterial effects.

**TABLE 3:** The diameter of the bacteriostasis circle of potential compounds.

Sample name	Bacteriostatic circle diameter/mm			
	*E. coil*	*S. aureus*	*P. aeruginosa*	*C. albicans*
AMC	—	25.55 ± 0.23	—	—
NFX	24.44 ± 0.37	—	25.14 ± 0.72	—
FLU	—	—	—	24.35 ± 0.53
Ethanol	—	—	—	—
ERCC	21.28 ± 0.43	22.78 ± 1.07	20.71 ± 0.36	21.13 ± 0.51
Chlorogenic acid	16.76 ± 0.87	18.36 ± 0.43	18.97 ± 0.74	16.71 ± 0.42
Theophylline	13.69 ± 0.66	16.23 ± 0.37	15.32 ± 0.27	15.31 ± 0.33
Gallic acid	13.45 ± 0.22	16.27 ± 0.73	14.13 ± 0.57	14.23 ± 0.43
Phloretin	19.18 ± 0.63	19.38 ± 0.27	20.11 ± 0.16	20.63 ± 0.10
Gallocatechin	15.22 ± 0.24	17.41 ± 0.55	17.42 ± 0.39	16.55 ± 0.35
Puerarin	14.91 ± 0.36	17.14 ± 0.32	16.43 ± 0.23	16.46 ± 0.42
Epigallocatechin	14.82 ± 0.18	17.24 ± 0.52	17.23 ± 0.65	15.37 ± 0.52

#### 3.4.2 The minimum inhibitory concentration of potential compounds

All test samples displayed antibacterial effects, as shown in Table 4. The MIC of ERCC and phlorizin against *E. coil, S. aureus, P. aeruginosa and C. albicans* were all 31.3 μg·mL^-1^ indicating superior bacteriostatic efficacy. And the MIC of theophylline and gallic acid against the four strains were 62.5 μg·mL^−1^, 125 μg·mL^−1^, 62.5 μg·mL^−1^, and 62.5 μg·mL^−1^, respectively. In addition, the MICs of four components, including gallocatechin, epigallocatechin, chlorogenic acid and puerarin, were in the range of 31.3–62.5 μg·mL^−1^ in that order.

**TABLE 4:** The minimum inhibitory concentration of potential compounds.

Sample name	MIC/µg·mL^-1^			
	*E. coil*	*S. aureus*	*P. aeruginosa*	*C. albicans*
ERCC	31.3	31.3	31.3	31.3
Chlorogenic acid	31.3	62.5	31.3	31.3
Theophylline	62.5	125	62.5	62.5
Gallic acid	62.5	125	62.5	62.5
Phloretin	31.3	31.3	31.3	31.3
Gallocatechin	31.3	62.5	62.5	31.3
Puerarin	31.3	62.5	62.5	31.3
Epigallocatechin	31.3	62.5	31.3	62.5

### 3.5 Determination of the contents of active ingredients from tea-seed oil by HPLC

#### 3.5.1 Calibration curves

Calibration curves were established by measuring the mixed standard solutions at seven different concentrations. The calibration curves presented in Table 5 show excellent linearity with high squared correlation coefficients (*R* ^2^ > 0.999) within the detected range.

**TABLE 5:** Calibration curves of seven compounds of the reference substance.

Sample name	Regression equation	*R* ^2^	Linearity Range(µg·mL^-1^)
Gallocatechin	Y = 2046.2X-8890.9	0.9996	0.75–15.00
Gallic acid	Y = 47808X-90789	0.9994	0.25–5.00
Epigallocatechin	Y = 2173.4X-12612	0.9996	0.75–15.00
Theophylline	Y = 37797X-75837	0.9992	0.25–5.00
Chlorogenic acid	Y = 14647X-55556	0.9993	0.25–5.00
Puerarin	Y = 17492X-47532	0.9997	0.25–5.00
Phloretin	Y = 36168X-66872	0.9994	0.25–5.00

#### 3.5.2 Method validation

**FIGURE 7:**
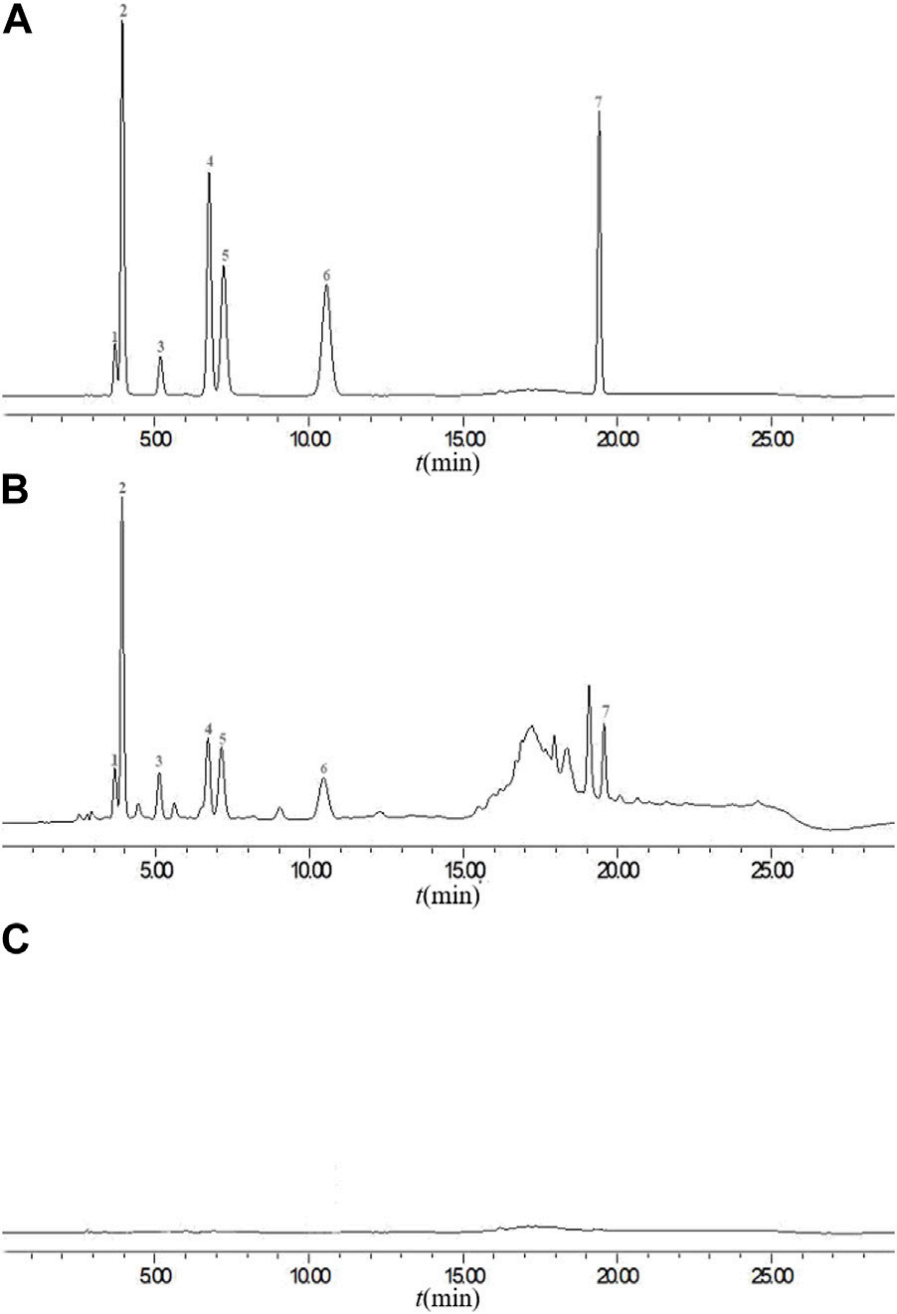
Chromatograms of the mixed standard solutions **(A)**, sample solution (YCZ-5) **(B)** and blank control **(C)**. 1: gallocatechin, 2: gallic acid, 3: epigallocatechin, 4: theophylline, 5: chlorogenic acid, 6: puerarin, 7: phloretin.

The precision, stability, and repeatability were tested and analyzed to validate the method. Under chromatographic conditions, an identical 10 µL mixed standard solution was injected six times consecutively and the RSDs were calculated. This methodexhibited a range of relative standard deviations (RSD) from 0.75% to 1.90%, indicating its appropriateness for quantitative analysis. In the stability trial, the mixed standard solution underwent testing at room temperature for various time intervals (0, 4, 6, 8, 10, 12, and 24 h). Based on the RSD values, the sample solutions demonstrated notable stability within a 24-h period, with values ranging from 1.5% to 1.9%. To ascertain the repeatability of the method, six independently prepared solutions from the same batch were analyzed. The RSD values for gallocatechin, gallic acid, epigallocatechin, theophylline, chlorogenic acid, puerarin, and phloretin were determined to be 0.46, 0.81, 0.34, 0.72, 1.30, 1.05, and 1.35%, respectively. According to the results, the proposed method exhibited a high level of repeatability. To verify the accuracy of the method, seven standard substances were precisely added to the TSO samples, and the samples were prepared using the above-mentioned method. In the peak area, the average recovery rate (n = 6) ranged from 99.91% to 102.35%, whereas the RSD ranged from 0.39% to 2.70%. Chromatograms of the mixed standard solutions (A), sample solution (YCZ-5) (B) and blank control (C) are presented in Figure 7.

#### 3.5.3 Content determination of ingredients

Among the 47 identified components, some of them have good antibacterial effects. For instance, gallocatechin (Zhao et al., 2021), gallic acid (Dibyajit et al., 2021), epigallocatechin (Sinsinwar and Vadivel, 2021), and other catechin derivatives have significant antibacterial effects, and can effectively inhibit bacteria and fungi. As a broad-spectrum antibacterial functional substance, chlorogenic acid possesses an inhibitory effect on a variety of pathogenic microorganisms (Wu et al., 2020). In addition, Puerarin (Fan et al., 2013), phloretin (Du et al., 2021) and theophylline (Shobha et al., 2016) have also been demonstrated to have certain antibacterial activity. Under chromatographic conditions, gallocatechin, gallic acid, epigallocatechin, theophylline, chlorogenic acid, puerarin, and phloretin were detected in ten kinds of TSO (Table 6). All ten TSO contained these seven compounds, but their concentrations varied greatly. Specifically, gallocatechin and epigallocatechin contents ranged from 5.27 μg·g^−1^–8.96 μg·g^−1^ and 4.37 μg·g^−1^–9.49 μg·g^−1^, respectively. Followed by gallic acid and theophylline with contents ranging from 1.48 μg·g^−1^–6.75 μg·g^−1^ and 1.43 μg·g^−1^–3.19 μg·g^−1^, respectively. The chlorogenic acid, puerarin, and phloretin contents were lower than 2.00 μg·g^−1^.

**TABLE 6:** The contents of the seven compounds in different kinds of TSO.

Sample no.	Content/μg·g^-1^						
	Gallocatechin	Gallic acid	Epigallocatechin	Theophylline	Chlorogenic acid	Puerarin	Phloretin
YCZ-1	8.96	2.73	9.49	1.43	0.40	1.49	0.41
YCZ-2	6.41	1.54	8.84	2.25	1.16	0.32	1.2
YCZ-3	6.86	5.24	4.37	2.18	1.98	1.21	0.33
YCZ-4	7.29	2.55	5.92	2.25	0.27	0.38	0.46
YCZ-5	7.06	1.4	6.69	3.19	0.76	0.77	0.51
YCZ-6	5.27	2.54	4.65	2.33	0.33	0.39	0.35
YCZ-7	7.2	6.75	5.61	2.28	0.32	0.32	0.38
YCZ-8	7.74	2.49	6.49	2.17	0.34	0.27	1.1
YCZ-9	5.85	1.48	5.72	2.24	1.17	0.26	0.25
YCZ-10	6.34	2.46	7.32	2.23	0.48	0.63	0.28

## 4 Discussion

*Camellia oleifera* Abel., a notable oilseed tree native to China, stands out in comparison to oil olives from the Mediterranean coast and oil palms from Southeast Asia. It is recognized as one of the three largest wooden oilseed tree species globally (Wu et al., 2020). The liquid oil derived from the seeds of *Camellia oleifera* Abel.*,* referred to tea seed oil (TSO), primarily consists of unsaturated fatty acids, with oleic acid comprising up to 80% and linoleic acid approximately 8% (Chang et al., 2020; Liu et al., 2021). Additionally, TSO is abundant in squalene, phytosterols, and vitamin E (Liu et al., 2022). Long-term consumption of TSO is beneficial to human health, as it is the highest-quality edible vegetable oil recommended by the World Health Organization (Wang et al., 2018). In addition to its edible value, TSO is commonly employd as a matrix excipient in cosmetic and pharmaceutical formulations due to its remarkable affinity for the skin and its capacity to facilitate drug penetration (Zhu, 2017). Additionally, TSO exhibits notable potential in inhibiting sebum secretion and improving the metabolic state of the skin (Li et al., 2010). Although clinical investigations have demonstrated the efficacy of topical TSO against diverse pathogenic microorganisms responsible for skin ailments, further research is warranted to elucidate its antibacterial constituents and underlying mechanisms (Zhang et al., 2022). In this study, UPLC-Q-TOF-MS, network pharmacology, and molecular docking were combined to explore the antibacterial functional ingredients in TSO that play an auxiliary role in its development.

The integration of environmentally sustainable principles in the process of product development enables the utilization of natural materials for the creation of novel pharmaceuticals and cosmetics (Espro et al., 2021). Moreover, natural products exhibit a commendable safety record and possess diverse biological properties (Burdock and Wang, 2017), thereby presenting prospects for enhancing the efficacy and safety of pharmaceuticals and cosmetics (Hill and Fenical, 2010). Nevertheless, the intricate nature of natural raw materials and the multitude of disease targets pose obstacles to the advancepment of drugs and drug-related products (Al-Awadhi and Luesch, 2020). Consequently, recent research endeavors have underscored the need for innovative methodologies to modify the existing paradigms of functional ingredient discovery and have enhanced our understanding of their mechanisms of action. In our study, the chemical constituents of TSO were identified through the utilization of UPLC-Q-TOF-MS. Network pharmacology and molecular docking techniques were concurrently employed to elucidate the potential bacteriostatic components and their underlying mechanisms of action in TSO. Subsequently, the antibacterial efficacy of seven potential active constituents was validated, and their concentrations in ten varieties of TSO were quantified. These discoveries hold significance for future investigations aimed at enhancing the pharmacodynamic properties of TSO through structural modifications of its active constituents.

The chemical compounds present in TSO have the potential to produce synergistic effects, leading to the identification of active antibacterial ingredients suitable for combination therapy (Zhang et al., 2022). In light of this, we have introduced the concept of ERCC, which is based on the average proportions of inhibitory components found in ten different types of TSO. Our aim is to investigate the antibacterial activity of ERCC and explore the synergistic effects of TSO’s chemical components. The results demonstrated that a homogeneous amount of ERCC had a better antibacterial effect than a single component, implying that multi-component action was more efficient.

This study has some limitations that must be acknowledged. Firstly, the complex structures of certain ingredients may have hindered their identification as components, and the TCM chemical database is continuously being enhanced and supplemented, thus potentially leading to unidentified ingredients. Furthermore, the lipophilic penetration-enhancing effect of TSO on the antibacterial effect and its underlying mechanism remain unclear. While this study presents encouraging findings, further research is necessary to elucidate the dose-effect relationship pertaining to the antibacterial efficacy of TSO and its ingredients.

## 5 Conclusion

This study employed UPLC-Q-TOF-MS to identify 47 chemical ingredients present in the methanol-extracted fractions of TSO. Notably, seven components, namely, gallocatechin, gallic acid, epigallocatechin, theophylline, chlorogenic acid, puerarin, and phloretin, demonstrated substantial inhibitory effects on *E. coli*, *S. aureus*, *P. aeruginosa,* and *C. albicans*. The underlying mechanism behind these effects is likely linked to the PI3K-AKT, estrogen, MAPK, and IL-17 signaling pathways. Moreover, the quantification of active ingredients in ten distinct varieties of TSO was conducted through the utilization of HPLC. The outcomes of this investigation possess the potential to expand our knowledge base regarding the utilization of TSO, furnish a theoretical framework for the exploration of antibacterial drugs and cosmetics derived from naturally occurring TSO, and establish a robust groundwork for the advancement and implementation of TOS products within clinical settings.

## Data Availability

The datasets presented in this study can be found in online repositories. The names of the repository/repositories and accession number(s) can be found in the article/supplementary material.
